# Melioidosis and the Heart: A Systematic Review

**DOI:** 10.3390/tropicalmed5030121

**Published:** 2020-07-23

**Authors:** Ragani Velusamy, Stephen Muhi

**Affiliations:** 1 The Royal Melbourne Hospital, Parkville 3050, Australia; ragani.velusamy@mh.org.au; 2 Victorian Infectious Diseases Service, Royal Melbourne Hospital, Parkville 3050, Australia

**Keywords:** melioidosis, cardiac, pericarditis, endocarditis, myocarditis

## Abstract

Melioidosis is caused by Gram-negative bacterium *Burkholderia pseudomallei*. Clinical presentation can vary from pneumonia, sepsis and multi-focal abscess formation. The aim of this study was to systemically review the cardiac manifestations of melioidosis in the literature and describe their epidemiology, microbiological diagnosis and outcomes. A systematic review of the peer-reviewed literature was carried out in PubMed and Google Scholar for human melioidosis cases with cardiac involvement. Quantitative data for cases of melioidosis were obtained, including age, sex, microbiological diagnosis, treatment, and outcome. 980 articles were screened, of which 31 articles were eligible. The most common cardiac site of infection was pericarditis, followed by endocarditis and myocarditis. Over 95% of cardiac involvement occurred in males, and mortality was the lowest in pericarditis and highest in myocarditis. Valvular vegetations were all small, left-sided, and did not require surgery. Antibiotic treatment included a bactericidal induction therapy with ceftazidime or a carbapenem ± TMP-SMX, followed by eradication therapy with TMP–SMX in most patients as previously established. In conclusion, melioidosis varies in clinical presentation and is also known as a great imitator. Although cardiac involvement is rare, this is the first systematic review to summarise all cases reported in the literature to date.

## 1. Introduction

Melioidosis was first characterised by Whitmore and Krishnaswami in 1912 as a glanders-like disease, yet easily distinguishable from glanders [1]. A century later, we now understand the causative agent for melioidosis to be *Burkholderia pseudomallei*, previously known as *Pseudomonas pseudomallei*. This Gram-negative organism is found abundantly in soil and water in endemic regions such as northern regions of Australia and South East Asia, although increasing reports have emerged in South America and South Asia. Humans acquire melioidosis through percutaneous inoculation or inhalation during exposure to contaminated soil or water [2].

The epidemiology and risk factors of melioidosis have been well described in the literature. Although known risk factors include diabetes mellitus, chronic liver or kidney disease, hazardous alcohol intake and immunosuppression, up to 16% of patients diagnosed have no identifiable risk factor [3]. The clinical presentation of melioidosis remains diverse, with acute, subacute, latent and disseminated infections reported. Prompt diagnosis is important, as higher mortality rates are associated with delayed commencement of appropriate treatment [4].

Cardiac involvement is reportedly rare, with an incidence of less than 1% [5,6]. Interestingly, in Whitmore’s original 1913 case series [7], he described a small vegetation on the mitral valve during post-mortem examination of a male morphia injector diagnosed with melioidosis. Although very few cases have apparently been reported in the subsequent century, there has been a notable increase in reports of melioidosis with cardiac involvement in the past decade. This is the first review which interrogates the available literature to better understand the cardiac manifestations observed in melioidosis. The aim of this study was to systemically review all published cases of culture proven *B. pseudomallei* with cardiac involvement, in order to describe epidemiological associations, underlying risk factors, treatment, and outcomes.

## 2. Materials and Methods 

### 2.1. Data Search

The Preferred Reporting Items for Systematic Reviews and Meta-Analyses (PRISMA) guideline [8] was utilised to conduct this systematic review. For identification of eligible studies, we performed a search in PubMed and Google Scholar with the following terms ‘melioidosis OR *Burkholderia pseudomallei* AND myositis OR myocarditis OR myocard OR pericarditis OR pericardial OR pericard OR tamponade OR endocarditis OR endocard.’ We also searched the grey literature using the Google search engine. The complete reference list accompanying all full text articles were scrutinised for relevant publications. The search included articles from the earliest published case of melioidosis until 9th May 2020.

### 2.2. Study Selection

Studies were included in the analysis if the following criteria were reported: (1) demographic and clinical characteristics; (2) microbiological data on *B. pseudomallei* diagnosis; (3) treatment and (4) documented cardiovascular outcomes. These included case reports, case series and letters, provided the above criteria were met. From the analysis, studies with the following criteria were excluded if (1) they did not report primary data and (2) did not involve humans. The titles and abstracts of the included studies were independently reviewed by two investigators (SM and RV). Full text publications were then screened for relevant articles and study selection was made based on consensus.

### 2.3. Study Outcomes

The primary study outcomes were to record: (1) epidemiology and risk factors of patients with melioidosis and cardiac involvement and (2) the site of cardiac involvement listed in the literature. Secondary outcomes included: (1) microbiological data, (2) treatment received and (3) mortality outcomes.

### 2.4. Data Extraction and Definitions

All eligible studies were analysed and data extraction was performed independently by two investigators (SM and RV). Extracted data included (1) study type; (2) year of publication; (3) country of *B. pseudomallei* acquisition; (4) patient demographics (age and sex); (5) environmental or occupational exposure to water or soil; (6) risk factors for melioidosis (diabetes, excessive alcohol use, chronic renal disease, chronic airways disease, malignancy, non-HIV related immunosuppression, smoking history, hypertension, coronary artery disease); (7) microbiological diagnosis; (8) valve and size of vegetation(s); (9) antimicrobial treatment and duration and (10) mortality outcomes. Grading of Recommendations Assessment, Development and Evaluation (GRADE) [9] criteria was used in the included studies to assess the quality of the reported evidence.

Given the rarity of *B. pseudomallei* endocarditis, we contacted the corresponding authors of all included studies of patients with endocarditis for additional information on echocardiographic findings, in order to better document the valvular vegetation size as well as demographic and clinical characteristics.

The largest case series of patients with *B. pseudomallei* pericarditis did not include any individual patient-level data [10], and, therefore, initially met exclusion criteria. Given the size of the case series and the otherwise rare nature of this condition, there was significant equipoise to request this data from the corresponding authors to obtain the individual patient data for study inclusion. 

## 3. Results

### 3.1. Literature Search

**Figure 1:**
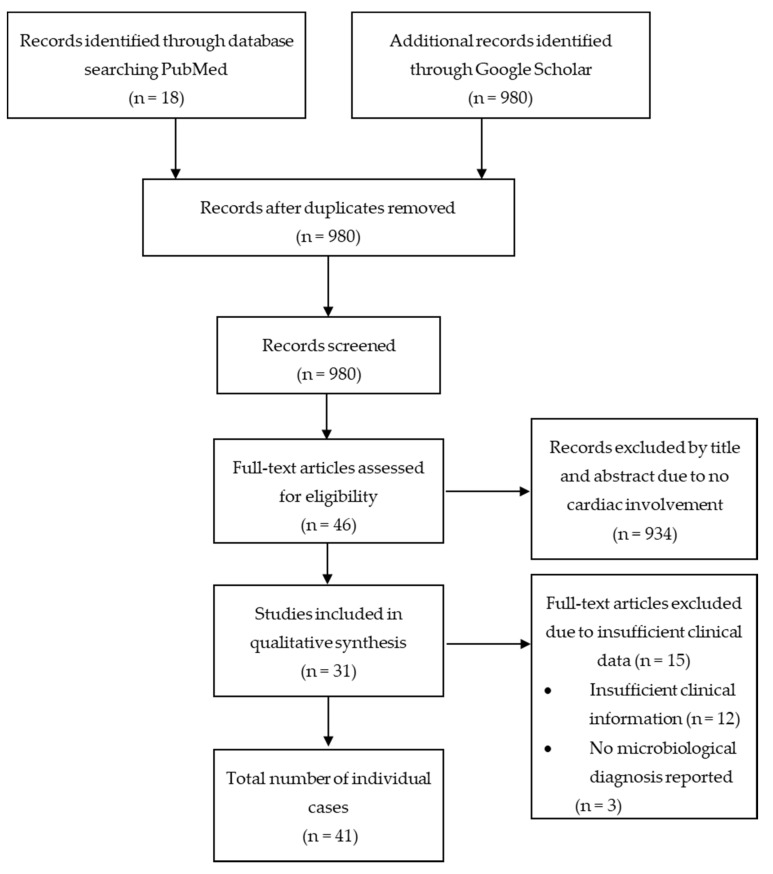
Preferred Reporting Items for Systematic Reviews and Meta–Analyses (PRISMA) flow chart.

We screened a total of 998 articles from PubMed and Google Scholar. After removing 18 duplicates, 980 articles were reviewed based on title and abstract. 46 articles with a total of 70 patients were selected for full text review. 15 articles, with a total of 29 patients were excluded due to insufficient clinical information (n = 12) [1,3,11,12,13,14,15,16,17,18,19,20] or no report of microbiological diagnosis (n = 3) [21,22,23]. A total of 31 articles [5,10,24,25,26,27,28,29,30,31,32,33,34,35,36,37,38,39,40,41,42,43,44,45,46,47,48,49,50,51,52] with 41 patients met the inclusion criteria of this systematic review. We did not exclude articles based on language, although only English-language articles were identified by our search strategy. Figure 1 shows a graphical representation of the literature search. 5,21,2.

### 3.2. Characteristics of Included Studies

The 31 studies included in this systematic review involved a total of 41 patients. Most of the studies were conducted in South East Asia (four in Thailand, five in Malaysia, three in each of Vietnam and Singapore) and South Asia (nine in India and two in Sri Lanka). There were also two studies from China and one study from each of Australia, Panama and Puerto Rico. In total, there were 22 case reports, six case series and three letters that made up the 31 studies. As per the GRADE criteria, the quality of studies was graded as low to very low [9].

### 3.3. Epidemiology, Microbiology, Treatment and Outcomes of Culture-proven B. pseudomallei with any Cardiac Involvement

The patients ranged from age 15 to 73 years, with a mean age of 58.8 years. The overwhelming majority (95.1%) of patients were male. The majority of patients (59.4%) had reported exposure to either soil or water, with 48.6% exposed to soil and 10.8% exposed to water. Soil exposure was classified as any exposure to soil including occupations such as farmer, lumberjack, landscaper, agriculturist, construction/renovator, military personnel while water exposure was classified as walking through a pond and occupations such as sailor or shipyard worker. Only 4% (2 out of 37 patients) were returned travellers from an endemic area. With respect to the known risk factors of melioidosis, diabetes was reported in 38.4% (15 out of 39 patients), coronary artery disease in 7.6% (3 out of 39 patients), smoker or ex-smoker in 20.5% (8 out of 39 patients), both hypertension and hazardous alcohol consumption in 7.6% each (3 out of 39 patients), both chronic kidney disease and malignancy each in 5.1% (2 out of 39 patients) and both airways disease and chronic liver disease each in 2.6% each (1 out of 39 patients). The most common microbiological diagnostic sample was pericardial fluid in 73.6% (28 out of 38 patients) followed by blood culture at 34.2% (13 out of 38 patients). As part of the induction therapy, 83.3% (20 out of 24 patients) received ceftazidime or a carbapenem, ± TMP–SMX. The mean duration of induction therapy was 4.6 weeks with a range between 0.85–29 weeks. For eradication, 55.6% (10 out of 18 patients) received TMP–SMX + doxycycline ± AMX–CLV. The mean duration of eradication therapy was 10.3 weeks with a range between 2.9 to 24 weeks. The overall mortality rate was 18.4% (7 out of 38 patients) with presence of extra-cardiac abscesses or septic emboli noted in 28.9% (11 out of 38 patients). The characteristics of patients with cardiac melioidosis are shown in Table 1.

### 3.4. Endocarditis

Whitmore was the first to describe endocarditis in melioidosis in 1913, reporting a 39–year–old male with small left-sided valvular vegetations post-mortem. In our systematic review, there were 11 patients with endocarditis reported in the literature [1,5,17,19,24,25,26,27,28]; six had adequate data for inclusion [5,24,25,26,27,28]. All six patients were males with an age range between 29 to 73 years and a mean age of 55.7 years. 50.0% (3 out of 6 patients) had reported exposure to soil. Both diabetes and smoking history (either current or ex-smoker) were each reported in 50% (3 out of 6 patients). 33.3% (2 out of 6 patients) had hazardous alcohol consumption while 16.7% each (1 out of 6 patients) had airways disease, hypertension or chronic kidney disease. A total of 83.3% (5 out of 6 patients) had positive blood culture for microbiological diagnosis while the remaining patient had a microbiological diagnosis from a liver aspirate. Regarding treatment, all patients were treated with ceftazidime or a carbapenem ± TMP–SMX as part of induction therapy. As part of eradication therapy, 75% (3 out of 4 patients) had either been treated with TMP–SMX ± doxycycline and 25% (1 out of 4 patients) was treated with only ciprofloxacin. The mortality rate was 16.7% (1 out of 6 patients) with 83.3% (5 out of 6 patients) demonstrating the presence of extra-cardiac abscesses or emboli. A total of 50% (2 out of 4 patients) had vegetations on the aortic valve while 50% (2 out of 4 patients) had vegetations on the mitral valve. The vegetation sizes for three patients were available and all were sub-centimere (1 × 1 mm, 3 × 8 mm and 2 × 3 mm). No patients underwent a valve replacement. The characteristics of patients with *B. pseudomallei* endocarditis are shown in Table 2.

### 3.5. Myocarditis

Only three patients with myocarditis were reported in the literature [29,47,48] and all were males with an age range between 38 to 58 years and a mean age of 48 years. 66.7% (2 out of 3 patients) had reported exposure to soil. The risk factors of diabetes and smoking history (either current or ex-smoker) were 50% each (1 out of 2 patients). The microbiological diagnosis was made through one of each modalities of bone marrow culture (33.3%), blood culture (33.3%) and histology (33.3%). A total of 66.7% (2 out of 3 patients) had extra-cardiac abscesses or emboli and all three patients with myocarditis died. The characteristics of patients with *B. pseudomallei* myocarditis are shown in Table 3.

### 3.6. Pericardial Involvement (Pericarditis or Suppurative Pericardial Effusion)

There were 49 patients with pericardial involvement reported in the literature [3,10,11,12,13,14,15,16,30,31,32,33,34,35,36,37,38,39,40,41,42,43,44,45,46,50,51,52,53], with sufficient information for study inclusion obtained to include 32 patients (78.0%) [10,30,31,32,33,34,35,36,37,38,39,40,41,42,43,44,45,46,50,51,52,53]. A total of 93.8% (30 out of 32 patients) were males with the age range between 15 to 73 years and a mean age of 47.5 years. A total of 68.4% (13 out of 19 patients) had reported exposure to soil whereas 21.1% (4 out of 19 patients) had reported exposure to water. 10.5% (2 out of 19 patients) were returned travellers from an endemic area. A total of 36.6% (11 out of 30 patients) had diabetes, 13.3% (4 out of 30 patients) were either current or ex-smokers, 10% (3 out of 30 patients) had coronary artery disease, 6.6% each (2 out of 30 patients) had hypertension, chronic liver disease or malignancy. Only 1 out of 30 patients had hazardous alcohol use reported. The microbiological diagnosis was made from pericardial fluid in 90.3% (28 out of 31 patients) in addition to either positive blood culture in 22.5% (7 out of 31 patients) or in sputum in 6.6% (2 out of 31 patients). For induction therapy, 81.2% (13 out of 16 patients) were treated with ceftazidime or a carbapenem ± TMP–SMX. The mean duration of induction therapy was 5.4 weeks with a range of 8 to 29 weeks. For eradication therapy, 46.7% (7 out of 15 patients) were treated with TMP–SMX + doxycycline ± AMX–CLV, 20.0% (3 out of 15 patients) were treated with ciprofloxacin + doxycycline + AMX–CLV and 33.3% (5 out of 15 patients) were treated with either TMP–SMX or doxycycline or sulfisoxazole. The mean duration of treatment was 14.5 weeks with a range of 2.9 to 24 weeks. The overall mortality rate was only 10.3% (3 out of 29 patients) and 13.7% (4 out of 29 patients) reported the presence of extra-cardiac abscess or emboli. A total of 20.7% (6 out of 29 patients) also underwent surgery with three patients receiving a pericardial window and three undergoing pericardiectomy. The characteristics of patients with *B. pseudomallei* pericarditis are shown in Table 4.

## 4. Discussion

Although the majority of patients with melioidosis present with an acute pulmonary infection [3], cardiac involvement is rarely reported. This is the first systematic review to assess cardiac involvement in patients with culture proven *B. pseudomallei* infection. The overwhelming majority of patients with cardiac involvement were males (95.1%). Although this is consistent with the male predominance worldwide, this is a higher percentage when compared to figures of 58.5% in Thailand to 84% in Singapore [53]. This study identified that the majority of patients were farmers, military personnel, agriculturists, construction or shipyard workers, so this may be due to males receiving disproportionately more occupational exposure to contaminated soil and water.

Diabetes was noted to be the most reported risk factor for cardiac involvement, followed by smoking history, with a mean age of 58.8 years. Diabetes is an important risk factor, given the global prevalence is 8.8%, increasing to 15% for the age group 55–59 years [54]. Pericarditis was the most commonly reported site of cardiac involvement, compared to endocarditis and myocarditis. Although this could be secondary to localised seeding of *B. pseudomallei* from contiguous pneumonia, only half of the patients (5 out of 10 patients) were noted to have either radiological or auscultatory evidence of pneumonia, raising the possibility of subclinical haematogenous seeding of the pericardium following primary infection, with subsequent reactivation, mimicking tuberculous pericarditis [10].

Acute melioidosis has a mortality rate of 20–50% worldwide, and over 50% in resource poor settings [55]. In comparison, this review found patients with cardiac involvement had an overall mortality of 18.4%, differing significantly depending on the site of cardiac involvement. Only 10.3% of patients with *B. pseudomallei* pericarditis died, compared to 16.7% of patients with endocarditis and 100% of patients with myocarditis, although most patients with myocarditis were diagnosed post–mortem. Patients with pericarditis may have had a lower than expected mortality rate, given 20.7% underwent surgery for pericarditis, which may have led to improved cardiac function, hence improving survival. Patients with endocarditis may be under-reported due to the small size of valvular vegetations, with all vegetations in this review reported as sub-centimetre, and none requiring valvular replacement. Additionally, the prolonged oral course of antibiotic therapy used for eradication may treat undiagnosed endocarditis.

Given the rarity of cardiac involvement, it is difficult to demonstrate any clear epidemiological association. Cardiac involvement was identified in all endemic areas, including reports from Panama and Puerto Rico in recent years. This may be secondary to global warming, leading to changes in weather in these regions and increasing the likelihood of infection [56]. 

The mainstay of microbiological diagnosis was culture from pericardial fluid or blood culture, using Ashdown or MacConkey agar in 85.7% (6 out of 7 patients), with one other diagnosis made using multilocus sequence typing. The treatment for melioidosis is well established, and usually includes a bactericidal induction therapy with ceftazidime or a carbapenem ± TMP–SMX, followed by eradication therapy with TMP–SMX. Duration of therapy lasts for months and is usually guided by disease severity and organ involvement. We found 79.1%, (19 out of 24 patients) were treated with ceftazidime or a carbapenem ± TMP–SMX. Out of these 24 patients, 55.6% (10 out of 18 patients) had TMP–SMX for eradication with doxycycline ± AMX–CLV. The average induction duration of treatment was 4.6 weeks with an average eradication period of 10.3 weeks. For induction, 77.8% (7 out of 9 patients) were treated with intravenous ceftazidime at 2 gram 8 hourly, with the remaining treated with a dose of 2 gram but at a frequency of either 6 hourly or 12 hourly.

There are several limitations to this systematic review. It comprised entirely of observational case reports and case series, with overall low to very low quality of evidence. The small number of patients makes it difficult to draw definite conclusions. In order to identify and include as many published cases as possible, we searched multiple databases and the grey literature, and both the article search and data extraction were performed in duplicate. We were also able to include previously unpublished data from the largest case series to-date, maximising the number of patients included in this review. In summary, although cardiac involvement is rare, this is the first systematic review to summarise the cases reported in the literature to date and may be useful for the clinician who encounters this condition.

## Figures and Tables

**Table 1:** Characteristics of 41 patients with culture proven *Burkholderia pseudomallei* with cardiac involvement: environmental or occupational exposure to water or soil, risk factors for melioidosis, microbiological diagnosis, treatment, presence of extra-cardiac abscesses or emboli and mortality outcomes. Values depict cases among patients with available data.

Characteristic	Value
Male, n (%)	39 out of 41 (95.1%)
Age range (in years)	15–73
Age, mean (in years)	58.8
**Risk Factors**	
Soil exposure ^†^	18 out of 37 (48.6%)
Water exposure ^#^	4 out of 37 (10.8%)
Returned traveller	2 out of 37 (5.4%)
Diabetes	15 out of 39 (38.4%)
Coronary artery disease	3 out of 39 (7.6%)
Airways disease	1 out of 39 (2.5%)
Smoker or ex-smoker	8 out of 39 (20.5%)
Hypertension	3 out of 39 (7.6%)
Hazardous alcohol	3 out of 39 (7.6%)
Malignancy	2 out of 39 (5.1%)
Chronic liver disease	1 out of 39 (2.5%)
Chronic kidney disease	2 out of 39 (5.1%)
**Microbiological Diagnosis**	
Pericardial fluid	28 out of 38 (73.6%)
Blood culture	13 out of 38 (34.2%)
Sputum	2 out of 38 (5.2%)
Aspirate from another organ (liver)	1 out of 38 (2.6%)
Histology	1 out of 38 (2.6%)
Bone marrow culture	1 out of 38 (2.6%)
**Treatment**	
Induction therapy	
Ceftazidime or a carbapenem ± TMP-SMX	20 out of 24 (83.3%)
Ceftazidime or a carbapenem + doxycycline	1 out of 24 (4.2%)
Ceftazidime or a carbapenem + tetracycline	1 out of 24 (4.2%)
Chloramphenicol + tetracycline	2 out of 24 (8.3%)
Duration range (in weeks)	0.85–29
Duration mean (in weeks)	4.6
Eradication therapy	
TMP-SMX + doxycycline ± AMX-CLV	10 out of 18 (55.6%)
Ciprofloxacin + doxycycline + AMX-CLV	3 out of 18 (16.7%)
TMP-SMX or ciprofloxacin or doxycycline or sulfisoxazole	7 out of 18 (38.9%)
Duration range (in weeks)	2.9–24
Duration mean (in weeks)	10.3
**Outcome**	
Presence of extra-cardiac abscesses or emboli	11 out of 38 (28.9%)
Coincident pneumonia	10 out of 30 (33.3%)
Mortality	7 out of 38 (18.4%)

† Includes soil exposure and occupations such as farmer, lumberjack, landscaper, agriculturist, construction/renovator, military personnel. # Includes walking through a pond and occupations such as previous sailor, shipyard worker.

**Table 2:** Characteristics of 6 patients with culture proven *Burkholderia pseudomallei* endocarditis. Values depict cases among patients with available data.

Characteristic	Value
Male, n (%)	6 out of 6 (100%)
Age range (in years)	29–73
Age, mean (in years)	55.7
**Risk Factors**	
Soil exposure ^†^	3 out of 6 (50.0%)
Diabetes	3 out of 6 (50.0%)
Airways disease	1 out of 6 (16.7%)
Smoker or ex-smoker	3 out of 6 (50.0%)
Hypertension	1 out of 6 (16.7%)
Hazardous alcohol	2 out of 6 (33.4%)
Chronic kidney disease	1 out of 6 (16.7%)
Microbiological diagnosis	
Blood culture	5 out of 6 (83.3%)
Aspirate from another organ (liver)	1 out of 6 (16.7%)
**Treatment**	
Surgical	
Extraction of infected pacemaker lead	1 out of 6 (16.7%)
Induction therapy	
Ceftazidime or a carbapenem ± TMP-SMX	6 out of 6 (100%)
Duration range (in weeks)	0.85–6
Duration mean (in weeks)	3.7
Eradication therapy	
TMP-SMX + doxycycline	3 out of 4 (75.0%)
Ciprofloxacin	1 out of 4 (25.0%)
Duration range (in weeks)	3–9
Duration mean (in weeks)	6.0
**Outcome**	
Presence of extra-cardiac abscesses or emboli	5 out of 6 (83.3%)
Coincident pneumonia	3 out of 10 (33.3%)
Mortality	1 out of 6 (16.7%)
**Location of Vegetation**	
Aortic Valve	2 out of 4 (50.0%)
Mitral Valve	2 out of 4 (50.0%)
**Location of Vegetation**	Size of Vegetation
Aortic	1 × 1 mm
Mitral	3 × 8 mm
Mitral	2 × 3 mm

† Includes soil exposure and occupations such as farmer, lumberjack, landscaper, agriculturist, construction/renovator.

**Table 3:** Characteristics of 3 patients with culture proven *Burkholderia pseudomallei* with myocarditis. Values depict cases among patients with available data.

Characteristic	Value
Male, n (%)	3 out of 3 (100%)
Age range (in years)	38–48
Age, mean (in years)	48
**Risk Factors**	
Soil exposure ^†^	2 out of 3 (66.7%)
Diabetes	1 out of 2 (50.0%)
Smoker or ex–smoker	1 out of 2 (50.0%)
**Microbiological Diagnosis**	
Bone marrow culture	1 out of 3 (33.4%)
Blood culture	1 out of 3 (33.4%)
Histology	1 out of 3 (33.4%)
**Treatment**	
Induction therapy	
Ceftazidime or a carbapenem + TMP–SMX	1 out of 2 (50.0%)
Cephalothin and tetracycline	1 out of 2 (50.0%)
**Outcome**	
Presence of extra–cardiac abscesses or emboli	2 out of 3 (66.7%)
Coincident pneumonia	2 out of 10 (20.0%)
Mortality	3 out of 3 (100%)

† Includes soil exposure and occupations such as farmer, lumberjack, landscaper, agriculturist, construction/renovator, military personnel.

**Table 4:** Characteristics of 32 patients with culture proven *Burkholderia pseudomallei* with pericardial involvement (pericarditis or pericardial effusion). Values depict cases among patients with available data.

Characteristic	Value
Male, n (%)	30 out of 32 (93.8%)
Age range (in years)	15–73
Age, mean (in years)	47.5
**Risk Factors**	
Soil exposure ^†^	13 out of 19 (68.4%)
Water exposure ^#^	4 out of 19 (21.1%)
Returned traveller	2 out of 19 (10.5%)
Diabetes	11 out of 30 (36.6%)
Coronary artery disease	3 out of 30 (10.0%)
Smoker or ex-smoker	4 out of 30 (13.3%)
Hypertension	2 out of 30 (6.6%)
Hazardous alcohol	1 out of 30 (3.3%)
Malignancy	2 out of 30 (6.6%)
Chronic liver disease	2 out of 30 (3.3%)
**Microbiological Diagnosis**	
Pericardial fluid	28 out of 31 (90.3%)
Blood culture	7 out of 31 (22.5%)
Sputum	2 out of 31 (6.5%)
**Treatment**	
Surgical	
Pericardiectomy	3 out of 29 (10.3%)
Pericardial window	3 out of 29 (10.3%)
Induction therapy	
Ceftazidime or a carbapenem ± TMP–SMX	13 out of 16 (81.2%)
Ceftazidime or a carbapenem + doxycycline	1 out of 16 (6.3%)
Chloramphenicol + tetracycline or TMP–SMX	2 out of 16 (12.5%)
Duration range (in weeks)	8c29
Duration mean (in weeks)	5.4
Eradication therapy	
TMP-SMX + doxycycline ± AMX–CLV	7 out of 15 (46.7%)
Ciprofloxacin + doxycycline + AMX–CLV	3 out of 15 (20.0%)
TMP-SMX or doxycycline or sulfisoxazole	5 out of 15 (33.3%)
Duration range (in weeks)	2.9–24
Duration mean (in weeks)	14.5
**Outcome**	
Presence of extra-cardiac abscesses or emboli	4 out of 29 (13.7%)
Coincident pneumonia	5 out of 10 (50.0%)
Mortality	3 out of 29 (10.3%)

† Includes soil exposure and occupations such as farmer, lumberjack, landscaper, agriculturist, construction/renovator, indigenous # Includes walking through a pond and occupations such as previous sailor, shipyard worker.
